# Antioxidant Potential of Sea Cucumbers and Their Beneficial Effects on Human Health

**DOI:** 10.3390/md20080521

**Published:** 2022-08-15

**Authors:** Abul Hossain, Deepika Dave, Fereidoon Shahidi

**Affiliations:** 1 Department of Biochemistry, Memorial University of Newfoundland, St. John’s, NL A1C 5S7, Canada; 2 Marine Bioprocessing Facility, Centre of Aquaculture and Seafood Development, Marine Institute, Memorial University, St. John’s, NL A1C 5R3, Canada

**Keywords:** sea cucumber, antioxidants, phenolics and polyphenolics, protein hydrolysates and peptides, polysaccharides, carotenoids

## Abstract

Sea cucumbers are considered a luxury food item and used locally in traditional medication due to their impressive nutritional profile and curative effects. Sea cucumbers contain a wide range of bioactive compounds, namely phenolics, polysaccharides, proteins (collagen and peptides), carotenoids, and saponins, demonstrating strong antioxidant and other activities. In particular, phenolic compounds, mainly phenolic acids and flavonoids, are abundant in this marine invertebrate and exhibit antioxidant activity. Protein hydrolysates and peptides obtained from sea cucumbers exhibit antioxidant potential, mainly dependent on the amino acid compositions and sequences as well as molecular weight, displayed for those of ≤20 kDa. Moreover, the antioxidant activity of sea cucumber polysaccharides, including fucosylated chondroitin sulfate and fucan, is a combination of numerous factors and is mostly associated with molecular weight, degree of sulfation, and type of major sugars. However, the activity of these bioactive compounds typically depends on the sea cucumber species, harvesting location, food habit, body part, and processing methods employed. This review summarizes the antioxidant activity of bioactive compounds obtained from sea cucumbers and their by-products for the first time. The mechanism of actions, chemical structures, and factors affecting the antioxidant activity are also discussed, along with the associated health benefits.

## 1. Introduction

Sea cucumbers are marine invertebrates that have been used for food, cosmetics, and traditional medicine. Around 100 species of sea cucumbers are harvested for commercial purposes, which have been widely consumed in Asian countries, including China, Indonesia, Japan, Korea, and Malaysia. Traditionally, sea cucumbers are a luxurious and nutritious food, and have been used to cure rheumatism, kidney problems, reproductive disorders, impotence, asthma, joint pain, back pain, hypertension, cuts and burns, wound injuries, and constipation [1]. In the contemporary market, various products originating from different body parts of the sea cucumbers are available. This mainly includes dry tablets made from the body wall, liquid extract prepared from whole sea cucumbers, and extracts obtained from the skin of sea cucumbers [2]. Nutritionally, sea cucumbers contain high levels of protein (40–60%), and low levels of lipids (mainly polyunsturated fatty acids (PUFAs)), minerals (e.g., calcium, zinc, iron, and magnesium), and vitamins (e.g., A, B1, B2, and B3) [3]. Apart from these, sea cucumbers contain a series of bioactive compounds, including triterpene glycosides (saponins), phenolics (flavonoids and phenolic acids), polysaccharides (fucosylated chondroitin sulfate), proteins (collagen and peptides), cerebrosides, and sphingoids, which demonstrate potential antioxidant, anticancer, anti-hypertension, anti-inflammatory, antithrombotic, anti-diabetic, anti-obesity, and antimicrobial activities [2]. However, biological activities mainly depend on the species, chemical structures, molecular weights, and testing methods. For example, fucosylated chondroitin sulfate of different sea cucumbers (*Stichopus tremulus*, *Pearsonothuria graeffei*, *Isostichopus badionotus*, and *Holothuria vagabunda*) exhibited potent anticoagulant activity, which could be associated with the sulfation pattern of the fucose branch [4]. Moreover, Hossain et al. [5] suggested that the antioxidant activity of sea cucumber phenolics is mostly linked with the nature of phenolic compounds, sea cucumber body parts, pre-treatment, and the assays used to determine the activity.

Antioxidants can prevent or slow down oxidative stress in cells and therefore play an important role in controlling various diseases, mainly cardiovascular ailments, cancer, and inflammatory diseases. Human bodies generate more reactive oxygen species (ROS) (e.g., hydroxyl radical, superoxide radical anion, and hydrogen peroxide) under stress. Therefore, a lack of enzymatic (e.g., glutathione peroxidase (GPx), superoxide dismutase (SOD), and catalase) and non-enzymatic antioxidants (e.g., α-tocopherol (vitamin E), ascorbic acid (vitamin C), carotenoids, glutathione, and phenols) ultimately leads to cell damage [6]. Apart from this, antioxidants are commonly used to improve the oxidative stability of food and food products, mainly those that are rich in lipids. As a result, the dietary inclusion of natural antioxidants is receiving growing interest and demand due to the potential carcinogenic effect of synthetic antioxidants. Sea cucumbers are one of the major marine animals containing various bioactive compounds with antioxidant activity. The purpose of this review is, for the first time, to summarize the antioxidant activity of bioactive compounds obtained from sea cucumbers and their by-products in order to categorize this echinoderm as a potential candidate in the functional food and nutraceutical sectors. Therefore, we report on the antioxidant activities and detailed mechanisms and chemical structures of the bioactive compounds of sea cucumbers along with their beneficial effects. The relevant data were collected from Scopus, PubMed, and ScienceDirect after searching different keywords such as “sea cucumber”, “antioxidant activity”, “bioactive compounds”, and “health benefits”, among others.

## 2. Bioactive Compounds of Sea Cucumbers and Their Antioxidant Activity

**Figure 1:**
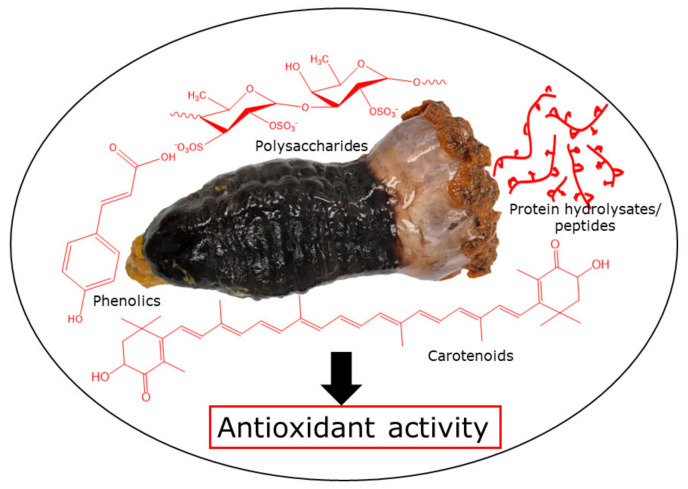
Sea cucumber antioxidants.

Sea cucumbers are a highly marketable echinoderm, which contains numerous bioactive compounds. These include proteins (collagen and peptides), polysaccharides, saponins, carotenoids, and phenolics with multiple biological and pharmacological properties, mainly antioxidant activity [2,7]. Antioxidants are substances that scavenge free radicals and hence prevent oxidation. The main mechanisms involved are hydrogen atom transfer (HAT), single electron transfer (SET), metal chelation, and reducing power. Therefore, the effectiveness of antioxidants in a specific medium is mainly dependent on the number and arrangement of the hydroxyl groups in the molecules of interest [8]. For example, phenolic antioxidants can donate hydrogen atoms from the hydroxyl groups to lipid radicals in order to neutralize the oxidation reaction, but the phenoxyl radicals that are produced are resonance stabilized and are therefore not involved in further oxidation, thus breaking the cycle of the generation of new radicals. Antioxidants with specific structures can also chelate metal ions (e.g., ferrous and copper), where metal ions can no longer act as an initiator of lipid oxidation due to the formation of a complex between the metal ions and antioxidants. Besides, synergistic effects can be observed among various antioxidants such as phenolics, α-tocopherol, β-carotene, and ascorbic acid [9,10]. Numerous techniques are available for determining antioxidant activity, including radical scavenging assays that include SET (e.g., ferric-reducing antioxidant power (FRAP), Trolox equivalent antioxidant capacity (TEAC), and the 2,2-diphenyl-1-picrylhydrazyl (DPPH) assay) and HAT (e.g., total radical-trapping antioxidant parameter (TRAP) and oxygen radical absorbance capacity (ORAC)) mechanisms [11,12]. However, each assay has a different mechanism of action, thus providing varied results for the antioxidant potential of the same sample [13]. On the other hand, due to lipid oxidation, the quality attributes of food, including flavor, color, and texture, deteriorate, which ultimately decreases the shelf life and nutritional value of food. Thus, antioxidants are widely used to control the rate and extent of lipid oxidation in foods. One of the main assays to measure the degree of lipid oxidation is the thiobarbituric acid (TBA) test that measures the TBA reactive substances (TBARS), which are then used to determine the secondary oxidation products, mainly the aldehydes, from among the others that are believed to produce rancid flavors and aromas [14]. Autoxidation is one of the main pathways of lipid oxidation in which PUFAs are involved in a free radical chain reaction under heat, light, or metal ions. Synthetic antioxidants such as butylated hydroxytoluene (BHT), butylated hydroxyanisole (BHA), tert-butylhydroquinone (TBHQ), and propyl gallate (PG) have been used as antioxidants in foods to prevent oxidation and off-flavor development. Nevertheless, due to the carcinogenic and toxicity characteristics of some synthetic antioxidants, researchers have shown much attention towards natural antioxidants [15,16]. For instance, sea cucumbers and their by-products are a good source of phenolic acids and flavonoids, which show strong antioxidant activity. The specific bioactive compounds in most common sea cucumbers and their antioxidant activities are detailed below (Figure 1).

### 2.1. Antioxidant Potential of Sea Cucumber Phenolics and Their Beneficial Effects on Human Health

Phenolic compounds are secondary metabolites that contain one or more aromatic rings and hydroxyl groups. Moreover, phenolic compounds play a key role in protecting plants by engaging in defense mechanisms against ultraviolet radiation, herbivory, and pathogen attacks. Phenolics are also involved in the plants for growth regulation and are responsible for the color, flavor, bitterness, and astringency of foods. Plant phenolics are mainly derived from phenylalanine and, in some cases, tyrosine. The formation of *trans*-cinnamic acid from phenylalanine is catalyzed by phenylalanine ammonia-lyase (PAL), whereas *p*-hydroxycinnamic acid from tyrosine is catalyzed by tyrosine ammonia-lyase (TAL) [17]. Phenolics can be categorized into different groups, namely phenolic acids, flavonoids, tannins, stilbenes, lignans, and coumarins [18]. Due to their antioxidant, antimicrobial, and coloring properties, phenolic compounds have received significant attention from several industries, especially from the food, pharmaceutical, cosmetics, packaging, and textile industries. In the food industry, phenolics are used as preservatives to inhibit the oxidation process and microbial growth of food products.

Plant- and marine-based phenolic compounds are receiving increased attention due to their potential health benefits and multiple biological activities. Most of the phenolics have so far been researched from the terrestrial environment, but less attention has been paid to the marine environment, even though it provides many healthy foods due to its biodiversity. Sea cucumbers are one of the marine invertebrates that serves as a possible source of phenolic compounds with strong antioxidant activity. This could be due to the absorption of phenolics from phytoplankton, the primary food source for sea cucumbers. Phytoplankton is a rich source of phenolic compounds, including phenolic acids, flavonoids, and tannins [2,19]. For example, Ceesay et al. [20] reported that sea cucumbers contain catechins and flavonols as they feed mainly on seaweeds, rich sources of flavonoids. Various species of sea cucumbers have different levels of phenolic compounds and varied antioxidant activities. This might be due to the different geographic locations, food habits, and harvesting times. Therefore, suspension-feeding species, such as *Cucumaria frondosa*, may have more phenolics when compared to the deposit-feeding species. Table 1 shows the phenolic compounds of different sea cucumbers and their antioxidant activities.

It has been reported that the different body parts of sea cucumbers, such as body wall, tentacles/flower, and viscera, contain a significant amount of phenolics with strong antioxidant activity. For example, Althunibat et al. [21] compared the antioxidant activity of three Malaysian sea cucumber species (*Holothuria leucospilota*, *Holothuria scabra*, and *Stichopus chloronotus*) without viscera, and reported that the extracts of *H. leucospilota* had higher total phenolic contents (TPC, 9.7 mg gallic acid equivalents (GAE)/g), but *H. scabra* contained a lower amount of TPC (1.53 mg GAE/g). *S. chloronotus* extracts showed a higher DPPH radical-scavenging capacity (80.58%) compared to the *H. scabra* (77.46%) and *H. leucospilota* (64.03%) extracts. Likewise, methanol extracts of *H. scabra* were found to be a good source of phenolics (30.52 mg GAE/g), dominated by 3- and 4-hydroxybenzaldehyde [22]. Wulandari et al. [23] cultured *Holothuria scabra* in an open pond system and found that antioxidant activity such as ABTS and hydroxyl radical-scavenging activities as well as ferric-reducing antioxidant power (FRAP) were related to the total flavonoid content (TFC). Besides, TPC and TFC were determined in the body wall of *H. leucospilota*, which contained 2,4-bis(1,1-dimethylethyl)-phenol [20]. Pre-treatments also affect the content of sea cucumber phenolic and their antioxidant activities. For example, free, esterified, and insoluble-bound phenolics from the body wall and internal organs of Atlantic sea cucumber (*Cucumaria frondosa*) were determined using high-pressure processing (HPP) pre-treatment [5,24]. Results demonstrated that HPP significantly improved the TPC, TFC, and antioxidant activity. TPC varied between 3.05 and 3.98 mg GAE/g for the body wall and 2.32–3.02 mg GAE/g for internal organs, while the TFC was 1.22–1.55 mg catechin equivalents (CE)/g and 1.01–1.24 mg CE/g for body wall and internal organs, respectively. Additionally, phenolic extracts obtained from the body wall and internal organs exhibited strong antioxidant activity in terms of DPPH, ABTS, and hydroxyl radical scavenging as well as metal chelation activities, which showed a strong positive correlation with TPC and TFC. Especially, TFC had a strong correlation with antioxidant activity, suggesting that sea cucumber phenolics are mostly flavonoids, which are responsible for the antioxidant effect. On the other hand, the antioxidant activity, TPC, and TFC were determined in extracts from different body parts (digestive tract, gonad, muscle, and respiratory apparatus) of *C. frondosa* [25]. The TPC varied from 0.22 to 2.36 mg GAE/g, while TFC ranged from 0.029 to 0.59 mg rutin equivalents (RE)/g and the oxygen radical absorbance capacity (ORAC) varied from 140 to 800 µmol Trolox equivalents (TE)/g. A higher TPC was also observed in the digestive tract when considering acetonitrile-rich fractions and ethyl acetate extracts, while the maximum TFC was obtained from the gonads using water-rich and acetonitrile-rich fractions. Similarly, Mamelona and Pelletier [26] determined the antioxidant activity (ORAC) of the viscera of *C. frondosa* using pressure liquid extraction (PLE) and observed that the ethanol extracts had higher ORAC values when compared to methanol, isopropanol, and water extracts at 60 °C extraction. Additionally, PLE allowed better extraction of TPC, total carotenoids, and α-tocopherol using ethanol followed by isopropanol, methanol, and water. In another study, the antioxidant property of fresh and processed *C. frondosa* with/without internal organs was evaluated [19]. The processed (rehydrated) samples, mainly those with internal organs, exhibited higher ORAC and DPPPH radical-scavenging activity, whereas fresh samples contained a significant amount of phenolics when compared to their rehydrated counterparts. However, Ridhowati et al. [27] reported that dried *Stichopus variegatus* contained a higher content of TPC (10.55–10.9 mg GAE/g) with strong DPPH radical-scavenging activity. Similarly, Husni et al. [28] stated that the TPC of *Apostichopus japonicus* body wall extract had a good correlation with antioxidant activity than the TFC, suggesting that phenolic compounds play an important role in exhibiting antioxidant activity.

**Figure 2:**
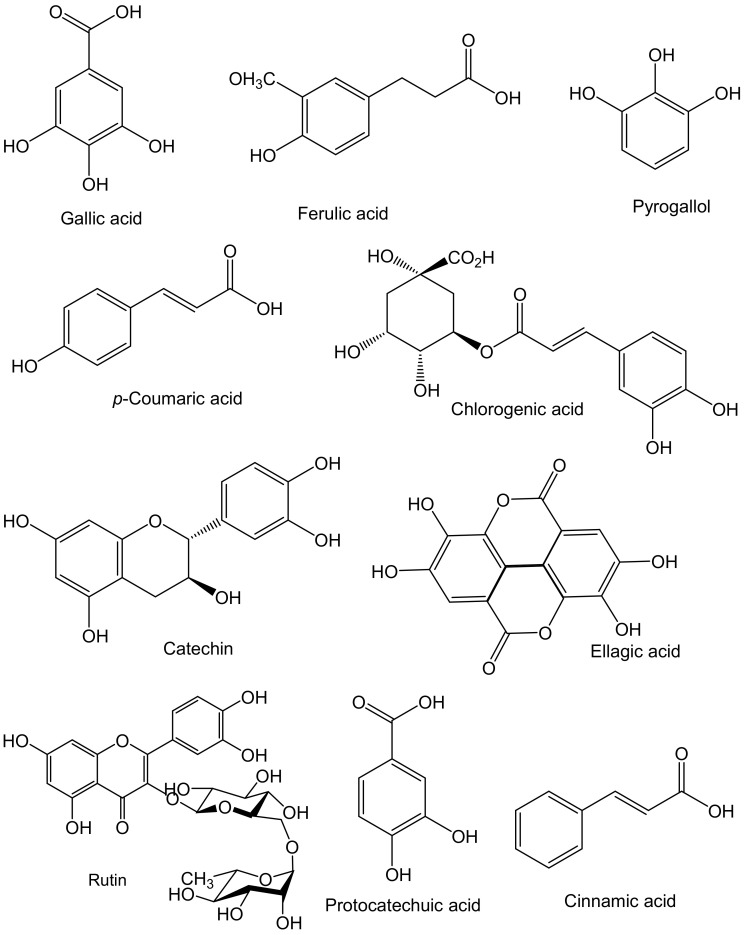
Major phenolic compounds found in sea cucumbers.

Sea cucumber phenolics are mainly phenolic acids and flavonoids. The most common phenolic compounds found in sea cucumbers are chlorogenic acid, gallic acid, *p*-coumaric acid, protocatechuic acid, ferulic acid, ellagic acid, cinnamic acid, catechin, rutin, quercetin, and pyrogallol (Table 2 and Figure 2).

For instance, high-performance liquid chromatography coupled with a mass spectroscopy (HPLC-MS) was used to identify the phenolic compounds of *Holothuria forskali* extracts, which were mostly phenolic acids such as quinic, gallic, rosmarinic, and salvianolic acids. The same study reported that quinic acid was abundant in different body parts, including the digestive tract, muscle, body wall, gonad, and respiratory tree, whereas only gallic acid and caffeoylquinic acid were present in the gonads of this sea cucumber [29]. Moreover, the body wall of *Holothuria atra* was an excellent source of chlorogenic acid (up to 92 wt%), and it also contained pyrogallol, coumaric acid, rutin, and catechin [30]. These phenolics demonstrated strong antioxidant activity, including DPPH radical-scavenging and metal-chelating activities. The presence of phenolics in the body wall could be due to the eating of phenolic-rich ingredients, mainly phytoplankton and particles derived from degrading marine macroalgae [5]. Likewise, phenolic compounds of *Holothuria atra* were identified and quantified using HPLC, and major compounds were chlorogenic acid (80.34%), pyrogallol, rutin, and coumaric acid [31]. Similarly, chlorogenic acid was the major component (~90%) in the body wall of *Holothuria arenicola*, whereas pyrogallol, rutin, and coumaric acid were also identified from this species [32]. On the other hand, Alper and Günes [33] prepared aqueous and methanolic extracts from *Holothuria tubulosa*, and HPLC analysis found epicatechin and 2.5 dihydroxybenzoic acid as the most abundant components, among others. 3-Hydroxybenzaldehyde and 4-hydroxybenzaldehyde were identified from the methanol extract of *Holothuria scabra*, which demonstrated strong antioxidant activity [22]. However, Hossain et al. [5] found that sea cucumber (*C. frondosa*) phenolics primarily existed in the free form followed by esterified and insoluble-bound phenolics. Moreover, HPP pre-treatment not only improved the content of individual phenolic compounds but also increased the number of phenolic compounds in their profile. UHPLC-QTOF-MS/MS identified 20 phenolic compounds from the body wall of *C. frondosa*, which are mainly phenolic acids and flavonoids such as protocatechuic acid, gallic acid, *p*-coumaric acid, ellagic acid, catechin, and epigallocatechin gallate. Similarly, 23 phenolic compounds were identified and quantified from the processing discards of *C. frondosa*; among them, 11 of which were identified for the first time in any species of sea cumber [24]. These were protocatechuic acid, homovanillic acid, gallic acid monohydrate, isoferulic acid, *p*-coumaroyl glycolic acid, sinapinic acid, caffeoyl glucoside, chicoric acid, *p*-hydroxycoumarin, scopoletin, and leachianol F.

**Table 1:** Phenolics in sea cucumbers and their antioxidant activity.

Species	Body Parts	TPC (mg GAE/g)	TFC (mg RE/g)	Antioxidant Assays					References
				DPPH (%)	ABTS (mg TE/g)	HRSA (mg TE/g)	MCA (mg EDTAE/g)	ORAC (mmol TE/g)	
*Holothuria forskali*	Dried sea cucumber (extract)	3.19–5.21	NA	NA	NA	NA	NA	NA	[34]
*Holothuria forskali*	Dried sea cucumber (hydroethanolic and aqueous extracts)	0.48	NA	1.06 ^f^	18.83 ^b^	NA	NA	NA	[35]
*Holothuria* *arguinensis*	Dried sea cucumber (hydroethanolic and aqueous extracts)	0.84	NA	0.13 ^f^	22.34 ^b^	NA	NA	NA	[35]
*Holothuria* *mammata*	Dried sea cucumber (hydroethanolic and aqueous extracts)	0.79	NA	0.31 ^f^	30.89 ^b^	NA	NA	NA	[35]
*Holothuria atra*	Body wall (phosphate buffer extract)	NA	NA	82 to 95	NA	NA	NA	NA	[31]
*Holothuria atra*	Dried sea cucumber (extract)	Detected	Detected	NA	NA	NA	NA	NA	[36]
*Holothuria arenicola*	Body wall (phosphate buffer extract)	NA	NA	82–95	NA	NA	NA	NA	[32]
*Holothuria scabra*	Dried sea cucumber (hexanes, ethyl acetate, and n-butanol extracts)	20–46.54	NA	NA	NA	NA	NA	NA	[37]
*Holothuria scabra*	Dried sea cucumber (methanol extract)	30.52	NA	33.77 ^c^	NA	NA	NA	NA	[22]
*Holothuria scabra*	Sea cucumber without viscera (aqueous and organic extracts)	1.53–4.85	NA	NA	NA	NA	NA	NA	[21]
*Holothuria scabra*	Dried sea cucumber (extracts)	2.02–2.86	0.35–2.49 ^e^						[23]
*Holothuria leucospilota*	Dried body wall (methanol, acetone, and water extracts)	4.58	0.84		NA	NA	NA	NA	[20]
*Holothuria leucospilota*	Sea cucumber without viscera (aqueous and organic extracts)	2.91–9.7	NA	3.91–5.44	NA	NA	NA	NA	[21]
*Cucumaria frondosa*	Body wall (acetone extract)	3.05–3.98	1.22–1.55	4.98–5.04 ^d^	7.51–8.01	10.47–10.65	0.41–0.53	NA	[5]
*Cucumaria frondosa*	Viscera (acetone extract)	2.32–3.02	1.01–1.24 ^a^	4.37–4.62 ^d^	7.36–7.87	9.57–9.85	0.29–0.44	NA	[24]
*Cucumaria frondosa*	Tentacles/flowers (acetone extract)	3.09	1.61	6.67 ^d^	NA	NA	0.55	NA	[38]
*Cucumaria frondosa*	Fresh and dried sea cucumber with/without viscera (methanol extract)	0.88–1.08	NA	4.51–7.48 ^b^	NA	NA	NA	2.09–2.6	[19]
*Cucumaria frondosa*	Dried digestive tract, gonads, muscles, and respiratory apparatus (extract)	0.22–2.36	0.029–0.59		NA	NA	NA	140–800 ^b^	[25]
*Stichopus variegatus*	Dried sea cucumber without viscera (aqueous extract)	10.55–10.9	NA	1.67–2.3 ^c^	NA	NA	NA	NA	[27]
*Apostichopus japonicus*	Dried internal organs (extract)	13.6–116.90	NA	NA	NA	NA	NA	NA	[39]
*Apostichopus japonicus*	Dried body wall (water and ethanol extracts)	18.65–40.99	5.92–30.38	3.2–16.37 ^b^	0.83–1.5 ^b^	NA	NA	NA	[28]
*Apostichopus japonicus*	Dried sea cucumber (methanol extract)	3.53–20.37	NA	NA	NA	NA	NA	NA	[40]
*Stichopus chloronotus*	Sea cucumber without viscera (aqueous and organic extracts)	1.66–8.27	NA	2.13 ^c^	NA	NA	NA	NA	[21]

Abbreviations are: NA, not available; HRSA, hydroxyl radical-scavenging activity; MCA, metal chelation activity; EDTAE, ethylenediaminetetraacetic acid equivalents; TE, Trolox equivalents; ^a^ data expressed as mg catechin equivalents/g; ^b^ data expressed as µmol TE/g; ^c^ data expressed as IC_50_ in mg extract/mL DPPH; ^d^ data expressed as mg TE/g; ^e^ data expressed as mg quercetin equivalents/g; and ^f^ data expressed as mg ascorbic acid equivalents/g.

**Table 2:** Phenolic compounds of sea cucumber.

Species	Body Parts	Identified Compounds (mg/100 g)	References
*Cucumaria frondosa*	Body wall	Protocatechuic acid (8.86), gallic acid (7.34),catechin (5.19), *p*-coumaric acid (5.11), epigallocatechin gallate (4.87), ellagic acid (4.55), hydroxygallic acid (3.9), *p*-hydroxybenzoic acid (3.66), *p*-coumaroyl glycolic acid (3.56), isoferulic acid (3.4), quercetin (3.35), *p*-hydroxybenzaldehyde (3.12), vanillic acid (3.04), cinnamic acid (2.9), syringic acid (2.51), myricetin (1.04), phlorizin (0.96), sinapinic acid (0.94), *p*-hydroxycoumarin (0.93), and caffeic acid (0.7)	[5]
*Cucumaria frondosa*	Viscera	Catechin (9.33), *p*-coumaric acid (7.15), protocatechuic acid (7.13), hydroxygallic acid (6.2), quercetin (5.48), gallic acid (5.66), chlorogenic acid (5.53), cinnamic acid (4.78), ellagic acid (4.33), syringic acid (4.23), *p-*hydroxybenzaldehyde (3.34), sinapinic acid (3.07), vanillic acid (3.05), caffeoyl glucoside (2.47), *p*-hydroxybenzoic acid (1.89), scopoletin (1.56), homovanillic acid (1.03), caffeic acid (1.01), *p*-coumaroyl glycolic acid (0.91), *p*-hydroxycoumarin (0.8), isoferulic acid (0.76), chicoric acid (0.73), and leachianol F (0.63)	[24]
*Cucumaria frondosa*	Tentacles/flower	Protocatechuic acid (6.91), catechin (6.32), gallic acid (6.14), *p*-coumaric acid (4.9), gallic acid monohydrate (4.46), quercetin (4.07), ellagic acid (3.86), cinnamic acid (3.35), sinapinic acid (2.56), syringic acid (2.51), *p-*hydroxybenzaldehyde (2.41), vanillic acid (2.4), chicoric acid (2.32), chlorogenic acid (2.25), caffeic acid (1.91), isoferulic acid (1.74), fraxin (1.64), kaempferol 3-*O*-glucoside (1.46), *p*-hydroxybenzoic acid (1.42), quercetin-3-*O*-arabinose (1.34), caffeoyl glucoside (1.05), epigallocatechin gallate (1.05), rosmarinic acid (1.05), scopoletin (1.04), homoveratric acid (1), ferulic acid (0.97), sinapine (0.97), homovanillic acid (0.97), *p*-coumaroyl glycolic acid (0.73), ferulic acid hexoside (0.72), and myricetin (0.2)	[38]
*Holothuria atra*	Body wall	Chlorogenic acid (80.34%), coumaric acid (2.43), pyrogallol (2.25%), and rutin (0.82)	[31]
*Holothuria atra*	Body wall	Chlorogenic acid (92.86%), pyrogallol (2.99%), rutin (1.83%), coumaric acid (1.55%), and catechin (0.51)	[30]
*Holothuria arenicola*	Body wall	Chlorogenic acid (89.66%), pyrogallol (1.88%), coumaric acid (1.23%), and rutin (1.06%)	[32]
*Holothuria scabra*	Dried sea cucumber	3-Hydroxybenzaldehyde and 4-hydroxybenzaldehyde	[22]
*Holothuria leucospilota*	Body wall	2,4-bis(1,1-dimethylethyl)-phenol	[20]
*Holothuria tubulosa*	Body wall	Epicatechin (790 µg/g), 2,5-dihydroxybenzoic acid (130.54–158.89 µg/g), ellagic acid (109.25–558.67 µg/g), gallic acid (133.16–205.87 µg/g)), chlorogenic acid, 3,4-dihydroxybenzoic acid, 4-hydroxybenzoic acid, vanillic acid, caffeic acid, *p*-coumaric acid, ferulic acid, cinnamic acid, rutin, naringin, and quercetin	[33]
*Holothuria forskali*	Digestive tract, muscle, body wall, gonad, and respiratory tree	Quinic acid (0.39–0.47 μg/mL), salvianolic acid (0.039–0.057 μg/mL), caffeoylquinic acid (0.13–0.14 μg/mL), caffeic acid, syringic acid, *trans* ferulic acid, *o*-coumaric acid, rosmarinic acid, and gallic acid	[29]

Sea cucumbers have been used as traditional medicine in Asian countries to treat hypertension, anemia, asthma, stomach ulcers, reproductive disorder, rheumatism, wound injuries, and constipation, among others [2]. However, various cell lines and animal studies have assessed the potential health benefits of bioactive compounds, mainly phenolics, of the sea cucumber. So far, the phenolics of the sea cucumber have been studied for their anticancer, antidiabetic, anti-inflammatory, antihypertension, anti-fibrotic, anti-aging, cardiovascular, and antioxidant properties. A summary of the potential health-promoting properties of sea cucumber phenolics is provided in Table 3.

For example, the anticancer properties of three Malaysian sea cucumbers (*H. leucospilota*, *H. scabra*, and *S. chloronotus*) extracts rich in phenolics were studied [21]. It was found that sea cucumber, mainly *S. chloronotus*, extracts prepared with organic solvents inhibited the growth of A549 (human non-small lung carcinoma) and C33A (human cervical cancer) cancer cells. Similarly, Alper and Günes [33] prepared aqueous and methanolic extracts of phenolics from *Holothuria tubulosa* in order to evaluate their cytotoxic effects. They suggested that phenolic extracts which were rich in epicatechin and 2,5-dihydroxybenzoic acid might inhibit the growth of cancer cells and induce apoptosis in A549 (human non-small lung carcinoma) and HeLa (cervix adenocarcinoma) cells. However, the phenolic compounds of the Atlantic sea cucumber’s (*C. frondosa*) body wall and internal organs were used in various in vitro biological assays [5,24]. Results suggested that phenolic compounds had the potential to inhibit DNA oxidation, low-density lipoprotein (LDL) oxidation, formation of advanced glycation end products (AGEs), and α-glucosidase and tyrosinase enzymes. The free phenolic fraction contained a significant number of phenolic acids and flavonoids, which showed strong inhibitory activity. On the other hand, Himaya et al. [40] suggested that phenolic compounds in ethyl acetate extracts prepared from *S. japonicus* may mediate the anti-inflammatory action via blocking of the MAPK (ERK and p38 MAPK) signaling pathway in murine macrophages. Likewise, the ethyl acetate fraction of *H. scabra* extracts, rich in phenolic compounds, inhibited pro-inflammatory cytokine synthesis at both the transcriptional and translational levels, mainly inducible nitric oxide synthase (iNOS), nitric oxide (NO), interleukin-1β (IL-1β), prostaglandin E_2_ (PGE_2_), and tumor necrosis factor-α (TNF-α) [37]. Carletti et al. [35] demonstrated that polyphenol-rich sea cucumber (*H. forskali*, *H. arguinensis*, and *H. mammata*) extracts, mainly hydroethanolic extracts, exhibited anti-inflammatory activity via COX-2 inhibition and osteogenic activity in a zebrafish (*Danio rerio*) model. However, phenolic-rich *Holothuria atra* extracts were used to determine the antioxidant efficacy against 7,12-dimethylbenz[a]anthracene (DMBA)-induced hepatorenal dysfunction [31]. It was found that the DMBA increased the level of liver malondialdehyde (MDA), decreased the level of glutathione-S-transferase (GST), and reduced glutathione (GSH), catalase (CAT), and SOD in the liver tissue in a rat model. Moreover, Fahmy [32] claimed that *Holothuria arenicola* extract was a good source of phenolic compounds with the potential to decrease the levels of total conjugated and unconjugated bilirubin and MDA as well as the activities of alkaline phosphatase and serum aminotransferases, and to increase GSH and serum albumin. Sea cucumber phenolics also showed antibacterial activities against various bacteria. For instance, Telahigue et al. [29] prepared ethyl acetate extracts from *Holothuria forskali* and tested them against *Bacillus cereus*, *Bacillus subtilis*, *Pseudomonas aeruginosa*, and *Escherichia coli*. It was found that ethyl acetate extracts are a good source of phenolic acids, which had the potential to inhibit *E. coli* and *B. subtilis*. Similarly, methanolic extracts of *Holothuria atra* contained phenolics with demonstrated antibacterial activities against *Pseudomonas aeruginosa* [36].

### 2.2. Antioxidant Potential of Protein Hydrolysates and Peptides and Their Health Benefits

Marine products and by-products are protein-rich and can be used to prepare protein hydrolysates, collagens, or peptides. The most common ways of producing protein hydrolysates include enzymatic (Flavourzyme, Alcalase, Protamex, papain, trypsin, chymotrypsin, and pepsin, among others), non-enzymatic (high-pressure processing, ultrasound, and supercritical fluids), organic solvents, and fermentation methods [41,42]. Among them, enzymatic procedures have received growing attention due to their higher efficiency, green nature, and lesser destruction than other techniques in order to produce value-added products for disease risk reduction and health promotion. In particular, microbial proteases, including Flavourzyme, Alcalase, and Corolase are favorable in industrial use due to their promising operational conditions [43]. However, the functionality of protein hydrolysates/peptides is mainly dependent on their amino acid compositions and sequences, molecular weight, and hydrophobicity/hydrophilicity, among others. Generally, bioactive peptides contain 3–20 amino acid units and show antioxidant activity [44]. Notably, the antioxidant activity of the bioactive peptides can improve in the presence of amino acids such as tyrosine, phenylalanine, proline, glutamic acid, histidine, and arginine. For example, proline is very common in collagen, shielding cells from the oxidation induced by free radicals [45].

Numerous studies have been performed on the preparation of protein hydrolysates from various marine sources, including sea cucumber, which demonstrate several health benefits. Protein hydrolysates of different sea cucumbers and their antioxidant activities are presented in Table 4.

The physicochemical and antioxidant properties of the protein hydrolysates of *C. frondosa* and its processing discards were evaluated using Alcalase, Flavourzyme, and Corolase as well as their combination [43]. The hydrolysates prepared with combination of enzymes were predominant in glutamic acid and displayed the highest radical-scavenging activity against ABTS and DPPH radicals as well as metal-chelation activity. In addition, hydrolysates were able to inhibit TBARS production in a meat model system and beta-carotene bleaching in an oil-in-water emulsion. They also noted that the level of free amino acids after hydrolysis, the degree of hydrolysis, amino acid sequence, and molecular weight played the main role in rendering radical-scavenging activity. Similarly, Yan et al. [46] prepared enzymatic hydrolysates from *C. frondosa* viscera using Alcalase, Flavourzyme, Neutrase, trypsin, papain, and bromelain and observed that Alcalase, Flavourzyme, and trypsin were major enzymes that resulted in strong antioxidant activity, possibly related to a high amount of hydrophobic amino acids in the hydrolysates. The choice of proteases in the preparation of protein hydrolysates plays a significant role in the resultant antioxidant potency and bioactivity of peptides. For example, Alcalase-produced protein hydrolysates obtained from *C. frondosa* exhibited up to 35% higher in vitro antioxidant activity (e.g., DPPH radical, hydroxyl radical, and superoxide radical anion-scavenging properties) than the trypsin-produced hydrolysates, suggesting that the amino acid composition and structural conformation of the peptides played main roles in determining antioxidant activity [47]. This is because trypsin-produced peptide fractions were not the same as Alcalase-produced peptide fractions, mainly when the sizes of the peptides were small (≤10 kDa). Moreover, in silico docking for in vivo function prediction demonstrated a better inhibitory activity in myeloperoxidase (a marker of in vivo oxidative stress) by Alcalase than by trypsin. Likewise, protein hydrolysates obtained from Atlantic sea cucumber viscera showed antioxidant activities in lipid oxidation tests and the ORAC assay, which could be related to the releasing of antioxidative peptides upon hydrolysis [48]. The findings of Wang et al. [49] suggest that *C. frondosa* internal organs hydrolysates have the potential to show anti-diabetic activity through insulin resistance and lipid metabolism syndromes. Besides, enzymatic hydrolysates prepared from *C. frondosa* by-products (aquapharyngeal bulb and internal organs) using nine different proteases, including Alcalase, bromelain, Flavourzyme, fungal protease, neutral protease, papain, peptidase AM (*Aspergillus melleus*), peptidase AO (*Aspergillus oryzae*), and Protamex, were tested against Herpes Simplex virus 1 (HSV-1) [50]. Results suggested that papain was the most efficient enzyme in demonstrating antiviral activities. Lin et al. [51] reported the anti-aging effect of sea cucumber (*C. frondosa*) hydrolysates, which was mainly linked to the low-molecular-weight (~<3 kDa) peptides. The anti-aging mechanism could be related to the up-regulated Klotho expression, down-regulated acetylcholinesterase activity, increased SOD and GSH-Px activities, and lipid and protein oxidation inhibition.

Antioxidant peptides were isolated from *Apostichopus japonicus* using papain, pepsin, trypsin, neutral protease, and acid protease, and it was found that trypsin-produced peptides exhibited the highest in vitro antioxidant activity (e.g., hydroxyl radical and superoxide radical anion-scavenging activities) when compared to the other enzymes [52]. Moreover, TP2b-1 was isolated as a novel antioxidative peptide showing radical-scavenging activity. Similarly, Zhu et al. [53] produced pepsin-solubilized collagen (PSC) from the body wall of *Apostichopus japonicus* and reported that PSC was more active in scavenging hydroxyl and DPPH radicals than the vitamins C and E, respectively. Likewise, low-molecular-weight gelatin hydrolysate was prepared from *Apostichopus japonicus* using Flavourzyme, which showed antioxidant activity against superoxide and hydroxyl radicals as well as inhibitory activity against melanin synthesis, tyrosinase enzyme, and melanogenesis [54]. However, Zhang et al. [55] purified peptides obtained from *Apostichopus japonicus* egg using ultra-filtration, gel filtration, and high-speed counter-current chromatography (HSCCC). The purified peptide was estimated to be about 30 kDa, which showed strong antioxidant activity against hydroxyl radicals. Recently, Guo et al. [56] determined the antioxidant and anti-aging effects of protein hydrolysate obtained from *Apostichopus japonicus* in vivo and characterized the function of peptides using a bioinformatics tool such as in silico. Results suggested that protein hydrolysate scavenged DPPH radicals directly and was able to inhibit ROS accumulation, reduce MDA content, and upregulate SOD activity in an animal model (*C. elegans*) under increased oxidative stress.

Sea cucumber-derived peptides were also reported to demonstrate a protective effect against UV-B-induced skin cell damage. Doungapai et al. [57] used papain, Alcalase, and Flavourzyme to prepare protein hydrolysate from *Holothuria scabra*; papain-derived hydrolysate had both hydrophilic and hydrophobic amino acids and showed strong UV-B protective activity in a HaCaT cell model. Rathnayake et al. [58] prepared *Holothuria spinifera* enzymatic hydrolysates (HSEA) using Alcalase, papain, Neutrase, α-chymotrypsin, and trypsin, where trypsin hydrolysate showed the greatest β-secretase inhibitory properties. RP-HPLC fraction of HSEA contained four bioactive peptides which reduced cellular levels of the β-secretase enzyme, amyloid-beta, and soluble amyloid precursor protein beta proteins as well as protecting SH-SY5Y (neuro-blastoma cell) from oxidation. On the other hand, microwave-assisted enzymatic (papain, Neutrase, pepsin, and trypsin) hydrolysis of collagen obtained from *Acaudina molpadioides* demonstrated antioxidant activity [45]. In particular, Neutrase-derived hydrolysate produced four bioactive peptides and showed strong DPPH radical-scavenging activity, suggesting that it was related to the presence of hydrophobic amino acids in their sequences and low molecular weights (<1 kDa). Similarly, collagen hydrolysates (molecular weight 14.4 to 25 kDa) obtained from golden sea cucumber revealed strong DPPH radical scavenging activity, where Neutrase was used to start the hydrolysis [59]. García et al. [34] designed novel functional food products using protein hydrolysate (Protamex and Alcalase) obtained from *Parastichopus tremulus* and *Holothuria forskali*, which demonstrated antioxidant, antihypertensive, and anti-inflammatory properties, especially those obtained from *H. forskali*. Furthermore, Safari and Yaghoubzadeh [41] suggested that *Holothuria leucospilata* protein hydrolysate with a molecular weight < 30 kDa exhibited strong antioxidant activity in in terms of the DPPH radical-scavenging assay and ferric reducing-antioxidant power, mainly those prepared from Alcalase rather than those from Flavourzyme. In addition, proteases were isolated from tentacles of the sea cucumber (*Isostichopus fuscus*) and then used to produce bioactive peptides from the body wall of *I. fuscus* [60]. It was found that the 3 kDa fraction obtained from the protein hydrolysate displayed a strong ORAC when compared to the high-molecular-weight (>3 kDa) fractions.

**Table 4:** Protein hydrolysates of sea cucumber and their antioxidant activity.

Species	Body Parts	Protein Hydrolysates/Collagens/Peptides	Antioxidant Assays					References
			DPPH (%)	ABTS (µmol TE/g)	HRSA (%)	MCA (µmol EDTAE/g)	ORAC (µmol TE/g)	
*Cucumaria frondosa*	Body wall, tentacles, and internal organs	Protein hydrolysates using Alcalase, Corolase, and Flavourzyme	7–14 ^a^	17.79–79.08	NA	16.5–37.43	NA	[43]
*Cucumaria frondosa*	Viscera	Protein hydrolysates using Alcalase, Neutrase, trypsin, papain, bromelain, and Flavourzyme	14.42	NA	27.04	NA	NA	[46]
*Cucumaria frondosa*	Viscera	Protein hydrolysates using Alcalase	NA	NA	NA	NA	421	[48]
*Isostichopus fuscus*	Body wall	Protein hydrolysates and peptides using proteases	NA	NA	NA	NA	0.00072	[60]
*Holothuria parvula*	Dried sea cucumber	Protein hydrolysates using Neutrase	5.25 ^b^	NA	NA	NA	NA	[59]
*Holothuria leucospilat*	Whole animal	Protein hydrolysates using Alcalase and Flavourzyme	35.3–68.27	NA	NA	NA	NA	[41]
*Holothuria scabra*	Dried sea cucumber	Protein hydrolysates using papain, Alcalase, and Flavourzyme	0.34–3.82 ^b^	1.28–1.65 ^b^	NA	NA	NA	[57]
*Acaudina molpadioides*	Body wall	Protein hydrolysates using papain, pepsin, trypsin, and Neutrase	~32	NA	NA	NA	NA	[45]
*Apostichopus japonicus*	Egg	Protein hydrolysates using papain and Flavourzyme	NA	NA	37–89.82	NA	NA	[55]
*Apostichopus japonicus*	Body wall	Collagen using pepsin	45.58	NA	~90	NA	NA	[53]
*Apostichopus japonicus*	Body wall	Protein hydrolysates Flavourzyme	NA	NA	0.28 ^b^	NA	NA	[54]

Abbreviations are: NA, not available; HRSA, hydroxyl radical-scavenging activity; MCA, metal-chelation activity; EDTAE, ethylenediaminetetraacetic acid equivalents; TE, Trolox equivalents; ^a^ data expressed as µmol TE/g; ^b^ data expressed as IC_50_ in mg/mL.

### 2.3. Antioxidant Potential of Sea Cucumber Polysaccharides

**Figure 3:**
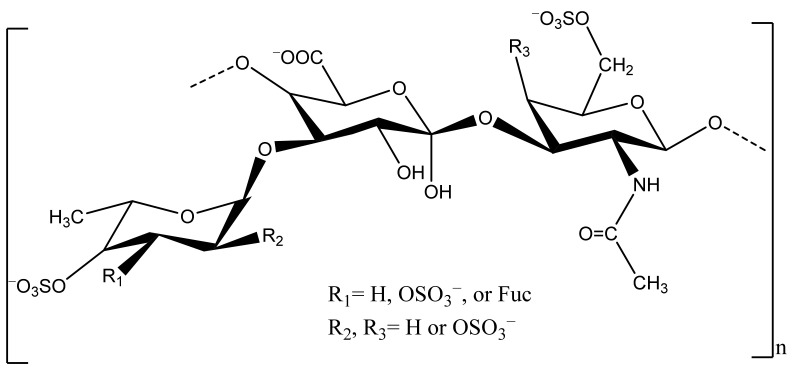
Chemical structure of FCS of sea cucumber.

Sea cucumber, mainly the body wall, is a good source of polysaccharides, including sulfated polysaccharides (fucosylated chondroitin sulfate, FCS) and fucan (Figure 3).

The chain conformation of polysaccharides mainly affects their antioxidant activity. In particular, the antioxidant activity of sulfated polysaccharides is associated with the molecular weight, degree of sulfation, type of main sugars, and glycosidic bonds. Hence, the antioxidant activity of sea cucumber polysaccharides is not related to a single factor but rather a combination of several factors. For example, Liu et al. [61] reported that the polysaccharides of *Apostichopus japonicus* were mainly composed of glucosamine, glucuronic acid, galactosamine, mannose, galactose, glucose, and fucose, which showed potent hydroxyl, DPPH, and superoxide radical-scavenging activities as well as reducing power. These could be due to the ability of free radicals to abstract anomeric hydrogen from polysaccharides [62]. Similarly, polysaccharides obtained from *Phyllophorus proteus* exhibited hydroxyl, DPPH, ABTS, and superoxide radical-scavenging activities, which may closely be linked to their structural features, including monosaccharide compositions and contents of their sulfate and carboxyl groups [63]. In particular, sulfate and carboxyl groups are extremely nucleophilic and may chelate metal ions (e.g., Cu^2+^ and Fe^2+^) and show hydroxyl radical-scavenging activity. Moreover, the sulfate group in sulfated polysaccharides could initiate superoxide radical-scavenging activity due to its electron-donating substituents in a saccharide ring [64]. However, Gao et al. [65] claimed that *Holothuria fuscopunctata* polysaccharides (fucan sulfate) had potent antioxidant activity for superoxide radicals, while exhibiting almost no scavenging effect for hydroxyl, DPPH, and ABTS radicals, which is related to the structural characteristics of sulfated polysaccharides. Moreover, Li et al. [66] found that *Stichopus chloronotus* fucoidan mainly consists of L-fucose and sulfate esters, which demonstrate significant inhibition of lipid peroxidation and immunoregulatory properties. The sulfate content, sulfate patterns, and molecular weight of fucoidan especially affect the inhibitory activities of superoxide radicals. Likewise, ultrasound treatment was found to slightly improve the antioxidant activity (DPPH radical-scavenging activity and ORAC) of fucoidan obtained from *Isostichopus badionotus* [67] which could be related to its lower molecular weight. Furthermore, Li et al. [68] stated that the sulfated polysaccharides of *Holothuria fuscogliva* show strong hydroxyl and superoxide radical scavenging activities as well as anticoagulant properties. On the other hand, the sulfation patterns of fucose branches of FCS obtained from Stichopus chloronotus, *Apostichopus japonicus*, and *Acaudina molpadioidea* were 4-*O*-, 2,4-di-*O*, and 3,4-di-*O*-sulfation, respectively, which inhibited DPPH and nitric oxide radicals as well as lipid peroxidation. The inhibitory activity may be affected by the sulfation patterns of the fucose branches. In this regards, 4-*O* sulfation residues exhibit the strongest antioxidant properties, while the opposite scenario is seen for 3,4-*O*-sulfated fucose residues [69]. Yu et al. [70] prepared fucoidan from *Thelenota ananas* and found a significant inhibitory effect of polysaccharides for superoxide radicals, closely related to its sulfate groups. Similarly, FCS isolated from *Acaudina molpadioidea* and *Holothuria nobilis* showed moderate antioxidant properties against hydroxyl, DPPH, and superoxide radicals in a dose-dependent manner. The presence of sulfate groups in polysaccharides with the ability to bind metal ions was found to be responsible [71]. In addition, sea cucumber (*Apostichopus japonicus*) gonadal polysaccharide exhibited DPPH and hydroxyl radical-scavenging activities as well as reducing power. The activity was greater in the presence of a higher content of sulfate groups and maintaining a lower molecular weight [72]. Besides, Qi et al. [73] suggested that sea cucumber processing liquor is mainly composed of mannose, glucose, and fucose, which showed strong DPPH, hydroxyl, and superoxide radical anion-scavenging activities. Moreover, in an in vivo model, these polysaccharides were able to increase the activity of catalase and SOD.

### 2.4. Antioxidant Potential of Carotenoids and Physiological Effects of PUFAs

**Figure 4:**
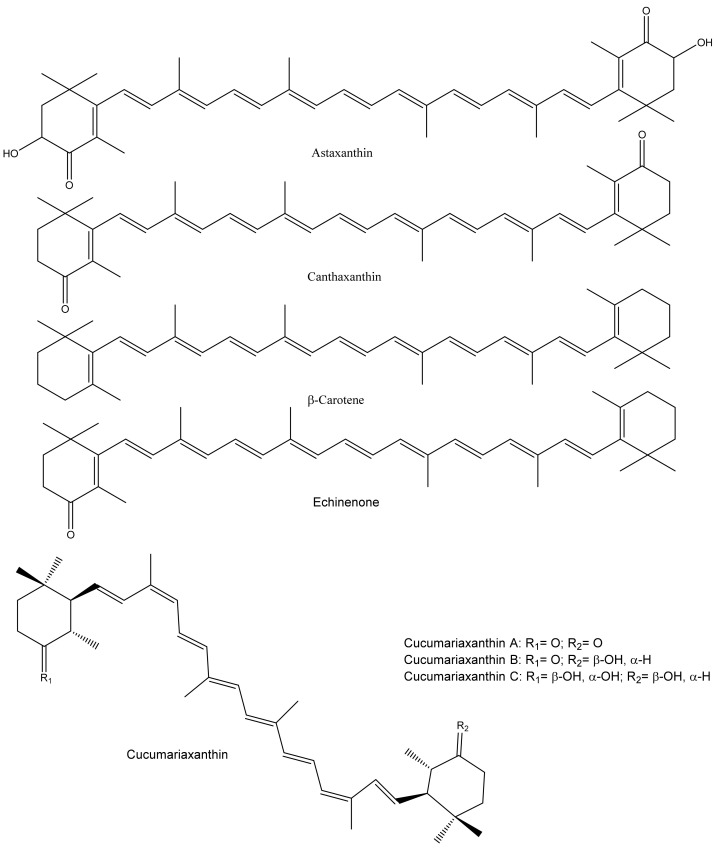
Major carotenoids found in sea cucumbers.

Marine animals, including sea cucumbers, are a good source of carotenoids that show structural diversity. Sea cucumbers, mainly their gonads and aquapharyngeal bulb/tentacles, contain various carotenoids (Figure 4).

The distinctive color of their internal organs is due to carotenoids that contain a series of conjugated C=C. Sea cucumbers, mostly passive feeder species, use algal or other carotenoid-rich materials as their primary food source, resulting in carotenoid accumulation in their tissues. The major carotenoids in sea cucumbers are astaxanthin and canthaxanthin, and their composition varies with species, geographical location, and body part. For example, Matsuno and Tsushima [74] investigated the carotenoid composition of seven sea cucumber species (*Stichopus japonicus*, *Holothuria moebi*, *Holothuria pervicax*, *Holothuria leucospilota*, *Cucumaria echinata*, *Cucumaria japonica*, and *Pentacta australis*), and β-echinenone, β-carotene, phoenicoxanthin, canthaxanthin, and astaxanthin were found in all of them. However, cucumariaxanthin A, B, and C were only present in *C. echinata*, *C. japonica*, and *P. australis*. Furthermore, Tsushima et al. [75] identified carotenoids (5,6,5′,6′-tetrahydro-carotenoids with 9Z,9′Z configurations) termed as cucumariaxanthins A, B, and C from *Cucumaria japonica* gonads, where cucumariaxanthin C showed antiviral activity on Epstein-Barr virus activation. Similarly, Maoka et al. [76] identified a new carotenoid (9Z,9′Z-tetrahydroastaxanthin) along with echinenone, canthaxanthin, β-carotene, astaxanthin, adonirubin, and cucumariaxanthin A from *Plesiocolochirus minutusalong*. Recently, 14 carotenoids were identified from the *Cucumaria frondosa japonica* Semper using supercritical CO_2_ extraction, where cucumariaxanthin and canthaxanthin were abundant [77]. In addition, the composition of fatty acids and carotenoids were analyzed from 12 sea cucumbers, and their effect on cancer cells was tested [78]. It was found that PUFAs, mainly eicosapentaenoic acid (EPA) and docosahexaenoic acid (DHA), and carotenoids significantly contributed to the cytotoxic activity against cervical (HeLa), colon (WiDR), and breast (T47D and MCF-7) cancer cells. However, little is known about the antioxidant activity of sea cucumber carotenoids and their detailed mechanisms. The antioxidant activity of these carotenoids could be related to the scavenging of ROS, mainly singlet oxygen (^1^O_2_) and peroxyl radicals, due to their special chemical features (conjugated C=C) [79].

Sea cucumbers, mostly their internal organs, contain around 3.5% lipids, mainly PUFAs (up to 50%) such as EPA (up to 30%) and DHA. Apart from this, sea cucumbers are rich in ether-phospholipids such as 1-alkenyl-2-acyl-phosphoethanolamine and 1-alkyl-2-acyl-phosphocholine [80]. They also reported that the most dominant PUFAs esterified to phospholipids were EPA and arachidonic acid (AA). Liu et al. [81] reported that freeze- and air-dried viscera of *C. frondosa* produced a similar composition of total fatty acid as fresh viscera, including a higher level of PUFAs (30–31%), mainly EPA (27–28%), and a lower content of omega-6 fatty acids (~1%). Therefore, the effect of sea cucumber PUFAs on various physiological functions is of interest. For example, EPA-enriched phospholipids (EPL) liposomes obtained from *C. frondosa* exhibited antitumor activity via the activation of caspase 9 and caspase 3 against S180 ascitic tumor-bearing mice [82]. Similar to this study, the impact of EPL isolated from *C. frondosa* on oxidative injury in rat pheochromocytoma (PC12) cells stimulated by tert-butylhydroperoxide and hydrogen peroxide was investigated [83]. Results suggested that the EPL was able to demonstrate a neuroprotective effect that could be related to inhibiting the mitochondria-dependent apoptotic pathway. Likewise, EPL obtained from *C. frondosa* could serve as a rapid regulator of fat burning due to its ability to increase the expression of genes related to β-oxidation in the epididymal adipose tissue and liver [84]. Furthermore, Hu et al. [85] stated that EPA-enriched phosphatidylcholine (EPC) isolated from *C. frondosa* significantly increased glycogen synthesis and insulin secretion as well as decreased blood glucose levels in diabetic rats. Moreover, reverse transcription-polymerase chain reaction (RT-PCR) analysis showed that EPC exhibited anti-hyperglycemic activities via up-regulating phosphoinositide 3-kinase (PI3K)/protein kinase B (PKB) signal pathway. On the other hand, long-chain bases from *C. frondosa* have the potential to regulate lipid metabolism via activation of the adenosine monophosphate-activated protein kinase (AMPK) pathway and inhibit adipogenesis via activation of WNT/β-catenin signaling [86]. Besides, Jia et al. [87] reported that glucocerebrosides of *C. frondosa* exhibit inhibitory effects on cell proliferation (HepG2 cells), which could be linked to the degree of saturation and/or hydroxylation of long-chain bases and fatty acids. In addition, fatty acids, mainly *cis*-9-octadecenoic acid and 1,3-dipalmitolein, of internal organs of *Apostichopus japonicus* showed potent α-glucosidase inhibitory activity [39].

### 2.5. Antioxidant Potential of Other Bioactive Compounds of Sea Cucumber

Apart from phenolics, protein hydrolysates/peptides, polysaccharides, and carotenoids, other bioactive compounds such as the saponins and cerebrosides of the sea cucumber also exhibit potential antioxidant activity. For example, the antioxidant and cytotoxic activities of organic and aqueous extracts of *Stichopus horrens* and *Holothuria edulis* were investigated and both extracts showed strong antioxidant activity against DPPH and linoleate (β-carotene bleaching assay) radicals. Meanwhile the organic extract showed higher cytotoxic effects against A549 (lung cancer) and TE1 (esophageal cancer) cells [88]. Moreover, four sulfated holostan-type triterpene glycosides (echinoside B 12-*O*-methyl ether, echinoside B, 24-dehydroechinoside B, and holothurin B) were isolated from the Saudi Red Sea cucumber *Holothuria atra*, which demonstrated antioxidant activity (reducing power and DPPH radical-scavenging activity) and cytotoxic effects against Ehrlich ascites carcinoma cells [89]. Furthermore, processing methods have a significant effect on antioxidant and physicochemical properties of sea cucumber products. For instance, sea cucumber powder (*Holothuria scabra*) was made using microwave heating, smoking, and steaming; the microwave treatment rendered a stronger DPPH radical-scavenging activity than the other two methods, possibly related to the heating via direct interaction with microwaved materials [90]. The antioxidant activity of microwave-treated powder could be linked to the presence of phenolics, steroids, and alkaloids. In addition, 21 Indonesian sea cucumber extracts were tested for their biological activities; *H. atra* and *S. vastus* extracts showed strong antioxidant (IC_50_ = 14.22 µg/µL) and antiviral activities, respectively [91]. Similarly, Rasyid et al. [92] analyzed the ABTS radical-scavenging activity of five Indonesian sea cucumber extracts, where *H. leucospilota*, *H. lessoni*, and *Stichopus quadrifasciatus* extracts had the strongest scavenging activity. In another study, Wulandari et al. [93] stated that *H. scabra* cultivated in the pond for 12 months demonstrated strong antioxidant and antibacterial activities. However, Wu et al. [94] determined the effect of *Acaudina molpadioides* cerebrosides on the tert-butyl hydroperoxide and hydrogen peroxide-induced oxidative damage in PC12 cells. The sea cucumber cerebrosides had a positive effect against oxidative damage by inhibiting mitochondria-mediated apoptosis, which may be related to their antioxidant effect.

## 3. Conclusions

Sea cucumbers, mainly *Holothuria atra*, *Holothuria scabra*, *Cucumaria frondosa*, and *Apostichopus japonicus*, are an excellent source of antioxidants such as phenolic acids, flavonoids, peptides, fucosylated chondroitin sulfate (FCS), fucoidan, and triterpene glycosides. Moreover, these compounds have the potential to show multiple biological activities, including anticancer, anti-inflammatory, anti-glycation, anti-tyrosinase, anti-hypertension, antithrombotic, anti-diabetic, and antimicrobial activities. Hence, sea cucumber antioxidants could serve as potential candidates in nutraceuticals, pharmaceuticals, cosmeceuticals, and functional foods. Nevertheless, further studies are required to understand their detailed chemical structures, mechanisms of action, and bioaccessibility and bioavailability through in vivo analysis and clinical trials to support the health claims and commercialize sea cucumber-derived value-added products.

## Figures and Tables

**Table 3:** Potential health promoting properties and mechanisms of action of sea cucumber phenolics.

Health Effects	Species	Body Parts	Responsible Compounds/Extracts	Results/Mechanisms	References
Anticancer	*Holothuria tubulosa*	Body wall	Aqueous and methanolic extracts rich in epicatechin and ellagic acid	Inhibited the growth of cancer cell lines and induced apoptosis in A549 (human non-small lung carcinoma) and HeLa (cervix adenocarcinoma) cells	[33]
	*Holothuria scabra*, *Holothuria leucospilota*, and *Stichopus chloronotus*	Sea cucumber without viscera	Aqueous extracts	Inhibited the growth of C33A (human cervical cancer) and A549 cancer cells	[21]
	*Stichopus variegatus*	Dried sea cucumber	Aqueous extracts	Possessed cytotoxicity on colon cancer cells WiDr, breast cancer cells T47D, and normal cells Vero	[27]
	*Holothuria scabra*	Dried sea cucumber	Extracts	Exhibited cytotoxic activity against human breast cancer cells (MDA-MB 231)	[23]
DNA oxidation inhibition	*Cucumaria frondosa*	Dried body wall and internal organs	Acetone extracts rich in phenolic acids and flavonoids	Inhibited hydroxyl and peroxyl radical-induced DNA oxidation	[5,24]
Anti-inflammatory	*Apostichopus japonicus*	Fresh sea cucumber	Ethyl acetate extract.	Inhibited the productions of NO (nitric oxide) and PGE_2_ (prostaglandin E_2_) by inhibiting iNOS (inducible nitric oxide synthase) and COX-2 (cycloxygenase-2)	[40]
	*Holothuria scabra*	Dried sea cucumber	Hexanes, ethyl acetate, and n-butanol extracts	Inhibited pro-inflammatory cytokine synthesis	[37]
LDL oxidation inhibition	*Cucumaria frondosa*	Dried body wall and internal organs	Acetone extracts rich in phenolic acids and flavonoids	Inhibited primary oxidation products, conjugated dienes (CD)	[5,24]
Hepatoprotective and curative	*Holothuria atra*	Body wall	Phosphate buffer extracts rich in chlorogenic acid	Alleviated the hepatorenal toxicity resulting from DMBA (7,12-dimethylbenz[a]anthracene) hydrocarbon exposure	[31]
	*Holothuria atra*	Body wall	Organic and aqueous extracts rich in chlorogenic acid	Exhibited hepatoprotective activity against thioacetamide-induced liver fibrosis in a rat model	[30]
Anti-cholestatic	*Holothuria arenicola*	Body wall	Phosphate buffer extracts rich in chlorogenic acid	Prevented liver damage following cholestasis	[32]
Antibacterial	*Holothuria atra*	Dried sea cucumber	Hexane, ethyl acetate, and butanol extracts	Showed inhibitory activity against *Pseudomonas aeruginosa*	[36]
	*Holothuria forskali*	Digestive tract, muscle, body wall, gonad, and respiratory tree	Ethyl-acetate extracts rich in quinic acid	*Escherichia coli* and *Bacillus subtilis* were inhibited	[29]
α-Glucosidase inhibition	*Apostichopus japonicus*	Dried internal organs	Organic extracts	Showed potential to inhibit α-glucosidase enzyme	[39]
	*Cucumaria frondosa*	Body wall	Acetone extracts rich in phenolic acids and flavonoids	Slowed down the activity of α-glucosidase enzyme	[5]
Antiglycation	*Cucumaria frondosa*	Dried body wall and internal organs	Acetone extracts rich in phenolic acids and flavonoids	Controlled the formation of advanced glycation end products(AGEs)	[5,24]
Anti-tyrosinase	*Cucumaria frondosa*	Dried internal organs	Acetone extracts rich in phenolic acids and flavonoids	Inhibited tyrosinase enzyme	[24]

## Data Availability

Not applicable.
